# Multi-Performance Evolution and Elasto-Plastic Damage Modeling of Basalt Fiber-Reinforced EPS Geopolymer Lightweight Concrete

**DOI:** 10.3390/polym17182471

**Published:** 2025-09-12

**Authors:** Feng Liang, Qingshun Yang, Jutao Tao

**Affiliations:** 1 Department of Civil Engineering, Qinghai University, Xining 810016, China; liangf0512@163.com (F.L.);15023466629@163.com (J.T.); 2 Key Laboratory of Building Energy-Saving Materials and Engineering Safety of Qinghai Province, Xining 810016, China

**Keywords:** LEGC, basalt fibers, EPS, elasto-plastic damage constitutive mode, mechanical properties, DIC-3D, CT, SEM

## Abstract

To elucidate the multi-performance evolution mechanisms of basalt fiber-reinforced lightweight expanded polystyrene geopolymer concrete (LEGC), a two-tiered investigation was conducted. In the first part, a series of LEGC mixtures with varying volume fractions of EPS (10–40%) and basalt fiber (BF) (0.4–0.8%) were designed. Experimental tests were carried out to evaluate density, flowability, compressive strength, flexural strength, and splitting tensile strength. Crack propagation behavior was monitored using DIC-3D speckle imaging. Additionally, X-ray CT scanning revealed the internal clustering of EPS particles, porosity distribution, and crack connectivity within LEGC specimens, while SEM analysis confirmed the bridging effect of basalt fibers and the presence of dense matrix regions. These microstructural observations verified the consistency between the synergistic effects of EPS weakening and fiber reinforcement at the microscale and the macroscopic failure behavior. The results indicated that increasing EPS content led to reduced mechanical strength, whereas the reinforcing effect of basalt fiber followed a rising-then-falling trend. Among all specimens, LEGC20BF06 exhibited the best comprehensive performance, achieving a compressive strength of 40.87 MPa and a density of 1747.6 kg/m^3^, thus meeting the criteria for structural lightweight concrete. In the second part, based on the experimental data, predictive models were developed for splitting tensile and flexural strengths using compressive strength as a reference, as well as a dual-factor model incorporating EPS and fiber contents. Both models were validated and demonstrated high predictive accuracy. Furthermore, a splitting tensile elasto-plastic damage constitutive model was proposed based on composite mechanics and energy dissipation theory. The model showed excellent agreement with experimental stress–strain curves, with all fitting coefficients of determination (R^2^) exceeding 0.95. These findings offer robust theoretical support for the performance optimization of LEGC and its application in green construction and prefabricated structural systems.

## 1. Introduction

Silicate cement-based concrete has emerged as the dominant construction material in civil engineering owing to its superior durability to traditional materials such as steel and timber. The versatility of the material in accommodating various structural sizes and shapes, the widespread availability of raw materials, and cost-effectiveness further contribute to its extensive application in construction projects [1]. These advantages render it an essential material in civil engineering.

However, concrete also exhibits certain drawbacks, including a high self-weight, susceptibility to early-stage cracking, and significant carbon emissions [2,3]. In 2020, worldwide production of Ordinary Portland Cement (OPC) amounted to 4.3 billion tons, experiencing an annual increase of roughly 9% [4]. The production of a single ton of OPC consumes approximately 1.5 tons of limestone and generates around 0.94 tons of CO_2_ emissions. Consequently, emissions from OPC production contribute to nearly 7% of global greenhouse gas emissions, raising serious environmental concerns [5,6]. Therefore, addressing the challenges associated with high weight and carbon footprint of concrete has become a key research priority. Adopting eco-friendly alternatives to OPC is increasingly being recognised as a promising approach.

To resolve these issues, researchers have explored lightweight expanded polystyrene geopolymer concrete (LEGC) as a potential solution to reduce both the carbon emissions and self-weight of conventional concrete [7,8,9]. Geopolymer concrete is an innovative and sustainable cementitious material that combines an alkaline activator with aluminosilicate materials. As an alternative to OPC, geopolymer concretes can incorporate various industrial by-products, including fly ash and slag, promoting resource recycling and significantly lowering CO_2_ emissions. Furthermore, integrating expanded polystyrene (EPS) particles as aggregates alleviates white pollution and substantially decreases the concrete density. Unlike traditional lightweight aggregates, EPS particles possess extremely low density, a closed-cell structure, and high deformability. Recent studies have further revealed that while EPS particles effectively reduce the overall density, they markedly compromise compressive and flexural strengths, with the extent of deterioration being closely dependent on particle size and dosage [10,11,12,13,14]. However, a known limitation of geopolymer concretes is their susceptibility to early-age shrinkage cracks and brittle failure. Studies have shown that incorporating fibres can mitigate these drawbacks, enhancing durability and mechanical properties. The incorporation of BF has been confirmed to enhance toughness, crack resistance, and post-peak energy dissipation, with the most pronounced effect observed at an optimal dosage of approximately 0.6%. Multiscale investigations further revealed that the bridging action of BF and the dispersion effect of EPS jointly regulate splitting tensile and flexural performances, exhibiting a nonlinear synergistic mechanism [15,16,17,18,19]. Basalt Fibre (BF) stands out among the various fibre types because of its exceptional chemical stability, superior mechanical performance, cost-effectiveness, and environmental benefits. These characteristics have led to their widespread use in concrete reinforcement [20,21,22].

Recent studies have demonstrated that while EPS particles are effective in reducing density, they markedly compromise compressive and flexural strengths, with the extent of deterioration strongly dependent on particle size and dosage [7,23,24]. In contrast, the incorporation of basalt fibers (BF) has been shown to improve toughness, crack resistance, and post-peak energy absorption, particularly at optimal dosages of approximately 0.6% [25,26]. Multiscale investigations further reveal that BF bridging and EPS dispersion collectively regulate splitting tensile and flexural performance, establishing a nonlinear synergistic mechanism [27]. These findings underscore the necessity of systematically evaluating EPS–BF geopolymer lightweight concrete from a mechanical perspective, thereby bridging the gap between macroscopic properties and microstructural mechanisms.

In summary, current research on fiber-reinforced LEGC has primarily focused on isolated mechanical properties, lacking a comprehensive evaluation of its multi-performance evolution and insufficiently addressing the coupling relationship between macroscopic behavior and microscale damage mechanisms. Moreover, existing constitutive models, often developed for traditional cement-based materials, fail to accurately capture the nonlinear damage evolution characteristics of LEGC under multi-variable interactions. To address these gaps, this study systematically investigates LEGC incorporating basalt fiber (BF) and EPS particles as a composite reinforcement system. A matrix of specimens with varying volume fractions of EPS (10–40%) and basalt fiber (BF) (0.4–0.8%) were designed to evaluate the coupled effects of EPS-induced weakening and BF-induced enhancement. A series of experiments, including density, flowability, flexural strength, compressive strength, and splitting tensile strength tests, were conducted to quantify performance variations under different mixture combinations.

In addition, DIC-3D imaging was employed to dynamically track the entire crack evolution process—from initiation and propagation to coalescence. X-ray computed tomography (CT) and scanning electron microscopy (SEM) analyses were further utilized to identify EPS particle agglomeration, pore-rich zones, and the bridging and anchoring behavior of basalt fibers, thereby establishing the consistency between macro- and micro-scale damage evolution.

To overcome the limitations of conventional constitutive models in characterizing the nonlinear response of LEGC, an elasto-plastic damage model was developed based on composite material theory and energy dissipation mechanisms. This model incorporates both a plasticity evolution term and a damage softening function, governed by six key parameters, to describe the full mechanical response from pre-peak hardening to post-peak softening. The model showed strong agreement with experimental stress–strain curves across all specimen groups. Furthermore, to enhance predictive capability and engineering applicability, a multivariate nonlinear regression model was proposed using compressive strength as a primary predictor. This model accurately estimates both splitting tensile and flexural strengths based on the EPS and BF contents, validating the dual-factor regulation mechanism. Collectively, this research establishes a robust framework for performance assessment and predictive modeling of LEGC, integrating macro-scale mechanical evaluation, microstructural analysis, and theoretical formulation. The findings provide valuable theoretical and practical insights for the design and application of LEGC in sustainable construction, energy-efficient buildings, and prefabricated structural systems.

## 2. Materials and Experimental Program

### 2.1. Raw Materials and Mix Design

**Figure 1:**
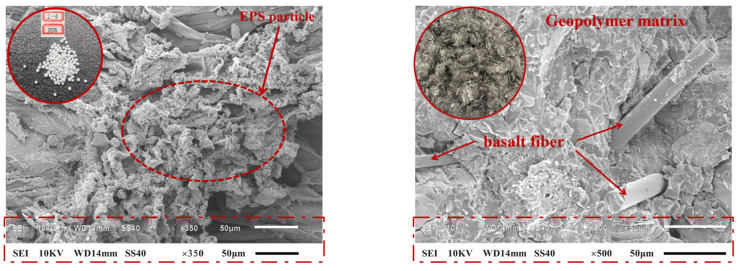
Appearance and microstructure of EPS particles and basalt fibers under SEM.

The fly ash used in this study was Class I fly ash obtained from Qinghai Leichang Trading Co., Ltd. (Qinghai, China).The Ground Granulated Blast Furnace Slag (GGBFS) complies with the GB/T 203 standard [28] and is classified as an S95-grade slag powder with a moisture content of 0.43% and a density of 3.1 g/cm^3^. Table 1 lists the compositions of the materials. The EPS particles used have a size range of 2–4 mm and a 16 kg/m^3^ density. The basalt fibres incorporated in this study were 9 mm long, and their morphologies and properties are illustrated in Figure 1 and summarised in Table 2. The alkaline activator consists of NaOH pellets and Na_2_SiO_3_ solution. NaOH pellets, with a purity of 98% (GR analytical grade), conformed to the GB/T 629-1997 standard [29] and were sourced from Xilong Scientific. The key parameters of the sodium silicate solution, supplied by Shanghai Zhecai IoT Co., Ltd.(Shanghai, China), are listed in Table 3. Uniformly graded natural river sand was used as fine aggregate in this study. Before testing, the acquired sand was dried inside an oven for 48 h at a temperature equal to 105 °C to achieve an SSD condition. Furthermore, a polycarboxylate-based high-performance water reducer accounting for 1.5% of the binder material weight was incorporated to enhance the performance of the EPS geopolymer concrete. It should be noted that the parameters listed in the table were obtained from the manufacturer’s technical datasheet.

The experiment was structured with the volume fractions of EPS particles and BF as the key variables. The material mix proportions were designed based on the method proposed by Jun et al. [3], as shown in Table 4. The notation LEGC20BF04 signifies that the EPS particle content was 20%, whereas the basalt fiber content was 0.4%.

### 2.2. Preparation and Curing of Specimens

**Figure 2:**
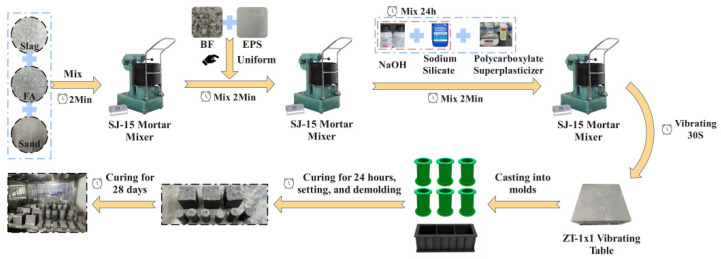
Process flow diagram of sample preparation.

The LEGC specimens were prepared following a sequential mixing procedure, as illustrated in Figure 2. Initially, fly ash, ground granulated blast furnace slag (GGBFS), and sand were dry-mixed in an SJ-15 mortar mixer for 2 min to obtain a uniform blend. Subsequently, pre-weighed expanded polystyrene (EPS) particles and basalt fibers (BF) were gradually added and uniformly dispersed by continuing the mixing for an additional 2 min. To mitigate the inherent tendency of untreated EPS particles—arising from their very low density relative to the surrounding matrix—to migrate upward and agglomerate, several measures were adopted: (i) a staged feeding and mechanical mixing process was applied to ensure that EPS particles were uniformly coated with the paste, thereby delaying segregation; (ii) basalt fibers were incorporated to form a three-dimensional restraining network that effectively limited EPS migration and clustering; and (iii) the relatively high viscosity of the alkali-activated geopolymer paste provided additional suspension capacity. Finally, a pre-prepared alkali activator solution, composed of sodium hydroxide (NaOH), sodium silicate (Na_2_SiO_3_), and a polycarboxylate-based superplasticizer and rested for 24 h, was introduced, followed by an additional 2 min of mixing until a homogeneous and workable consistency was achieved.

The fresh geopolymer concrete was poured into two types of ABS plastic moulds: cylindrical (50 mm × 100 mm) and rectangular prism (40 mm × 40 mm × 160 mm), as summarized in Table 5.

The cylindrical specimens (three per group, 39 in total) were designated for splitting tensile strength tests, while the rectangular specimens (three per group, 39 in total) were used for flexural and compressive strength tests. All moulds were placed on a ZT-1 × 1 vibrating table for 30 s to eliminate entrapped air and improve the compactness of the specimens. After 24 h of initial setting at room temperature, the specimens were demoulded and subsequently transferred to a curing chamber maintained at 22 ± 2 °C with a relative humidity of 50% ± 5%, where they were cured for 28 days to ensure sufficient strength development prior to mechanical testing.

### 2.3. Experimental Program and Testing Methods

#### 2.3.1. Physical Properties Testing

According to ASTM C138-16 [30], the fresh concrete density must be measured using at least three specimens to ensure accuracy, and the average of these measurements is reported as the final density value. In this study, cylindrical specimens with dimensions of Ø 50 × 100 mm were used for the density tests. In addition, the flowability of the fresh geopolymer paste was assessed in accordance with ASTM C1437-20 [31] using the standard flow table method. The paste was placed into a conical mold, and upon removal of the mold, the flow table was dropped 25 times. The maximum spread diameters along two orthogonal directions were measured, and their average was recorded as the final flow value. Each batch was tested three times, and the mean of the three readings was used to represent the flowability of the mixture.

#### 2.3.2. Mechanical Properties Evaluation

**Figure 3:**
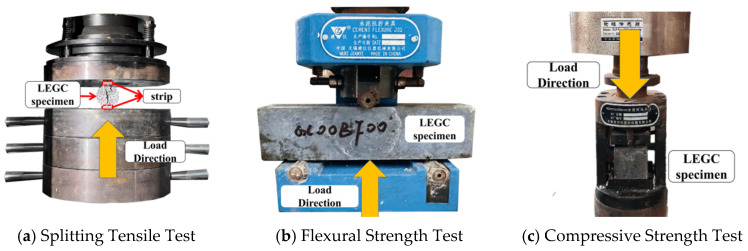
Testing Apparatus.

The mechanical performance tests in this study mainly included splitting tensile strength, flexural strength, and compressive strength evaluations. The corresponding test setups are illustrated in Figure 3. Splitting tensile tests were carried out using a microcomputer-controlled electrohydraulic servo testing machine (Model YAW-4306, MTS Systems (China) Co., Ltd., Shanghai, China) equipped with a 3000 kN load cell, following the ASTM C496-17 [32] standard. A stress-controlled loading mode was adopted with a loading rate of 0.02 MPa/s. To ensure uniform stress distribution across the specimen, bearing strips were placed between the loading platens and the cylindrical specimen ends. A high-speed camera was employed to capture the entire failure process. Each group consisted of three specimens, and the average value was reported as the splitting tensile strength.

Flexural strength tests were conducted using a CFJ-9 cement flexural test fixture manufactured by Wuxi Jianyi Instrument Machinery Co., Ltd. (Wuxi, China)., mounted on a loading frame with a 10 kN load cell, in accordance with ASTM C348-21 [33]. The tests were also performed under a stress-controlled mode with a loading rate of 45 N/s. Three prismatic specimens were tested for each group, and the average value was recorded as the flexural strength result.

Compressive strength tests were performed according to ASTM C349-18 [34], using the residual halves of the specimens previously subjected to flexural testing. A dedicated cement compression machine equipped with a 300 kN load cell was employed, operating at a loading rate of 2400 ± 200 N/s. The specimens were loaded on their lateral faces with a designated loading area of 1600 mm^2^. The load was applied continuously until specimen failure, and the peak load was recorded for calculating the compressive strength.

#### 2.3.3. Microstructural Characterization

After completion of the mechanical tests, small fragments approximately 3–5 mm in size were cut from the fracture surfaces of the specimens and prepared for scanning electron microscopy (SEM). Following gold sputter-coating, the microstructural morphology was examined using a scanning electron microscope (JSM-6610LV, JEOL Ltd., Tokyo, Japan) operated at an accelerating voltage of 10 kV with a secondary electron detector. In addition, X-ray computed tomography (CT) analysis was performed on the damaged cylindrical specimens (Ø 50 × 100 mm) using the nanoVoxel-0 series 3D tomography system (Sanying Precision Instruments Co., Ltd., Tianjin, China). The scans were conducted under a tube voltage of 150 kV, a current of 150 mA, and an exposure time of 0.5 s, with a voxel resolution of 41.525 μm and a 360° rotational range in cone-beam transmission mode. The 2D projections were reconstructed using ArtFDK (integrated in Thermo Scientific Avizo™ 9, Thermo Fisher Scientific, Bordeaux, France) software based on the filtered back-projection (FBP) algorithm, with beam hardening correction and median filtering applied to minimize artifacts. Quantitative analysis was subsequently carried out using Avizo9.5.0 software to obtain the pore size distribution, pore volume fraction, and fiber volume fraction, thereby enabling comprehensive characterization of the internal microstructural features.

## 3. Macroscopic Crack Evolution and Micromechanical Damage Mechanisms of LEGC

### 3.1. Digital Image Correlation (DIC) Analysis

**Figure 4:**
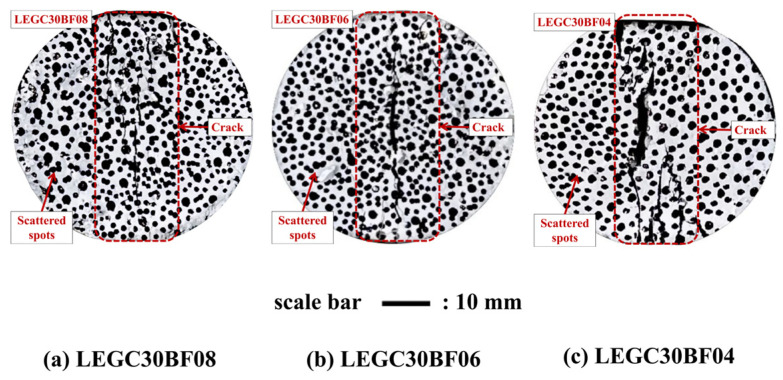
Final damage pattern of split tensile test at 30% EPS particle content.

Figure 4 illustrates the final crack patterns of LEGC specimens containing 30% EPS particles under splitting tensile loading, with varying BF contents of 0.4%, 0.6%, and 0.8%. Regardless of the BF dosage, a dominant central crack penetrating through the specimen was observed in all cases. Notably, the width of this main crack visibly decreased with increasing BF content, indicating that basalt fibers provided an effective bridging constraint during the early stages of crack initiation. This bridging effect helped to inhibit crack propagation and delay the overall failure process.

Moreover, as the applied load increased, secondary microcracks began to emerge adjacent to the main crack. The formation and expansion of these secondary cracks were significantly suppressed due to the synergistic effect of EPS particles and basalt fibers, highlighting their combined role in regulating crack evolution and improving the failure mode of the composite.

**Figure 5:**
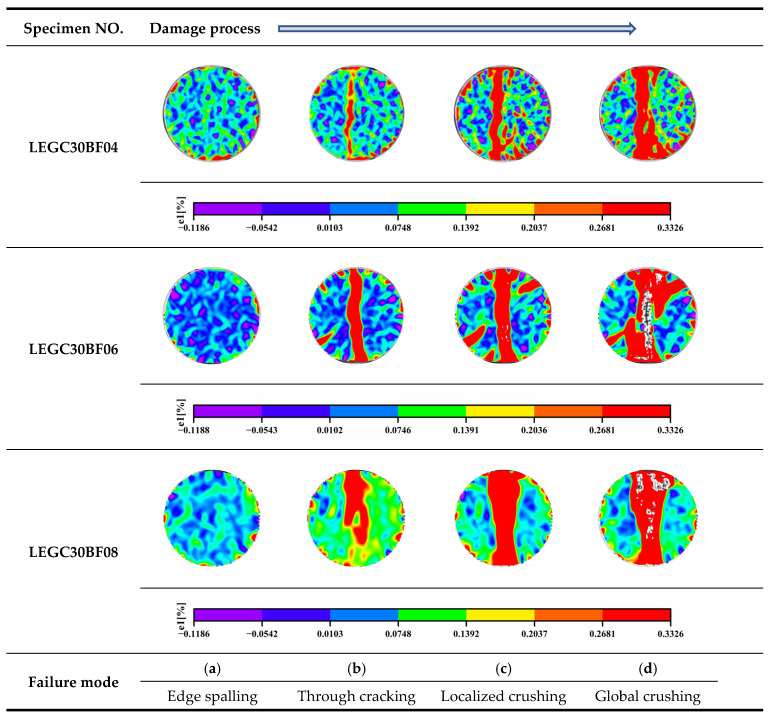
Crack development analysis in LEGC30 using DIC-3D scattering technology.

Cracking and failure in LEGC under splitting tension were monitored using DIC-3D. Using the LEGC30 series (BF = 0.4, 0.6, 0.8%) as a representative case, Figure 5 documents a four-stage evolution: (a) edge spalling with low background strain (blue–green) and no through-crack; (b) through-cracking, where a continuous high-strain vertical band forms—narrowest in LEGC30BF04, wider in LEGC30BF06, and widest in LEGC30BF08 with broader yellow/green halos, indicative of earlier coalescence of the main crack path; (c) localized crushing, during which the red damage field expands around the crack—largest in BF08, intermediate in BF06, and comparatively confined in BF04; and (d) global crushing, characterized by high-strain regions over most of the section and distinct whitish void-like streaks/patches in BF06/BF08 that are scarce in BF04.

The observed image features reflect the complete fracture process, evolving from microcrack initiation and propagation to final localization and overall crushing, accompanied by fiber bridging, occasional rupture or pull-out, as well as EPS interfacial debonding and local shear failure. Notably, the bright streaks observed in BF06/BF08 specimens are consistent with SEM/CT results, indicating that when the BF content reaches ≥0.8%, fiber clustering and elevated ITZ porosity are more likely to occur, thereby promoting the formation of interconnected pore networks that facilitate crack coalescence and accelerate damage accumulation. Overall, the DIC results demonstrate that, during crack penetration and crushing, the sequence of damage band width and failure severity follows the order BF04 < BF06 < BF08.

Moreover, as the EPS content increases from 10% to 40%, the crack propagation mechanism shifts from deflection-dominated multiple cracking with delayed coalescence to through-crack formation with rapid localization. At low EPS levels (~10%), the good matrix continuity facilitates crack initiation at matrix/ITZ flaws, followed by deflection around EPS beads; the crack path is tortuous with noticeable branching, which delays coalescence. At moderate EPS contents (~20%), EPS-induced weakening and fiber bridging are relatively balanced; cracks mainly deflect or slide along the ITZ, and microcracks gradually merge into a main crack at a reduced propagation rate. When the EPS content reaches ~30%, the increase in porosity and weak ITZ regions promotes the formation of a dominant through-crack and earlier localization along pore-rich bands. At high EPS levels (~40%), an interconnected pore–ITZ network governs the failure path, accelerating macroscopic instability. Fiber bridging is effective in narrowing the main crack and delaying coalescence at low to moderate EPS levels; however, when porosity and clustering effects dominate (i.e., at high EPS or excessive fiber content), the toughening effect approaches an upper bound.

### 3.2. Scanning Electron Microscopy (SEM) Analysis

**Figure 6:**
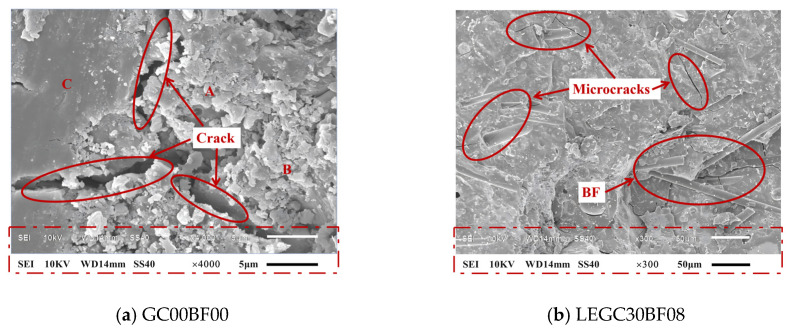
SEM images of selected specimens.

Figure 6a,b show the typical microstructural characteristics of the samples. A comparative analysis of the GC00BF00 and LEGC30BF08 specimens revealed that combining basalt fibres and EPS particles inhibited crack propagation, shifting the material failure mode from brittle to ductile.

Figure 6a shows the microstructure of the baseline sample GC00BF00. SEM analysis indicates that several unreacted FA particles, marked as “A”, are embedded within the geopolymer gel matrix. The structure labelled “B” represents the depolymerised structure formed by the alkali activation of the fly ash-slag system, exhibiting morphological features typical of low-crystallinity calcium silicate hydrate gel. The structure marked as “C” corresponds to the homogeneous geopolymer gel, which serves as the main matrix phase and aligns with the microstructural characteristics of geopolymer mortars reported in the literature [35].

**Figure 7:**
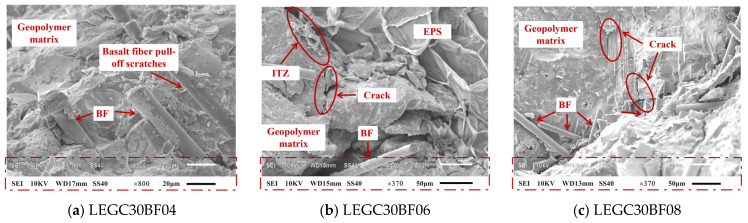
SEM images of specimens with 30% EPS particle content at different volume fractions of basalt fibres.

The results of the micromechanical analysis, as shown in Figure 7a, indicate that when the BF volume content is 0.4%, a practical bond-slip effect between the BFs and the matrix is not established. The fracture morphology reveals that the average fibre embedment depth reaches 80 μm. In this case, the primary resistance to crack propagation arose from the fibre-fracture-induced crack-bridging energy-dissipation mechanism.

As the BF volume fraction increased to 0.6%, the fibres exhibited an anisotropic yet well-dispersed distribution and formed a three-phase continuous load-bearing system comprising the geopolymer matrix, basalt fibers (BF), and EPS particles together with their interfacial transition zone (ITZ), as shown in Figure 7b. At this stage, SEM observations confirm that the fibers effectively bridge and anchor within the matrix and mechanically interlock with the ITZ around EPS, enabling all three phases to participate in load transfer through an interconnected stress-transfer network. In other words, fiber–matrix bonding, interfacial shear at the EPS–matrix interface, and matrix load sharing together establish uninterrupted load paths, which enhance crack bridging, delay crack coalescence, and improve fracture toughness. Consequently, the mechanical properties and toughness of the LEGC specimens were significantly enhanced, demonstrating a typical multiscale synergistic strengthening effect with notable improvements compared to the reference group. The so-called multiscale synergistic effect refers to a set of mutually reinforcing mechanisms operating across different length scales. At the macroscale, DIC results reveal a more ductile failure response at the optimal BF content of 0.6%. At the microscale, SEM observations confirm the development of a three-phase continuous load-bearing network (matrix–fiber–EPS/ITZ), which provides effective crack bridging and anchorage. At the mesoscale/3D pore level, CT analysis demonstrates that BF contents exceeding 0.6% (e.g., 0.8%) result in fiber clustering and the formation of pore-through channels, thereby disrupting matrix continuity.

As shown in Figure 7b, the LEGC30BF06 specimen after the compressive test exhibits a typical porous and fractured morphology. The relatively high EPS content (30%) weakens the bonding between the particles and the geopolymer matrix. During loading, debonding initiates at the interface, and the ITZ becomes a stress concentration zone, generating microcracks that propagate along the interface to form distinct voids. With increasing stress, the continuity of the matrix skeleton is further reduced, and at peak stress the matrix fails along these microcracks, resulting in a loose and porous structure. Moreover, although 0.6% basalt fibre provides crack-bridging capacity and partially suppresses crack propagation, many fibres were pulled out or were surrounded by fractured matrix during compression, leading to voids and clustered cracks. Finally, the fracture surface contains numerous irregular pores and cracks, originating from EPS particle rupture and spalling as well as debris detachment caused by energy release during loading.

However, when the BF content reached 0.8%, as shown in Figure 7c, the SEM analysis indicated the formation of fibre clustering, which reduced the effective load-bearing volume fraction of the matrix. This clustering led to a significant increase in the pore volume within the interfacial transition zone, and notable stress concentrations occurred at the crack tip, resulting in transgranular fracture of the matrix. Consequently, the macroflexural strength of the specimens is significantly diminished. In general, the observed micro-failure mechanisms aligned well with the experimental results.

### 3.3. X-Ray Computed Tomography (CT) Analysis

**Figure 8:**
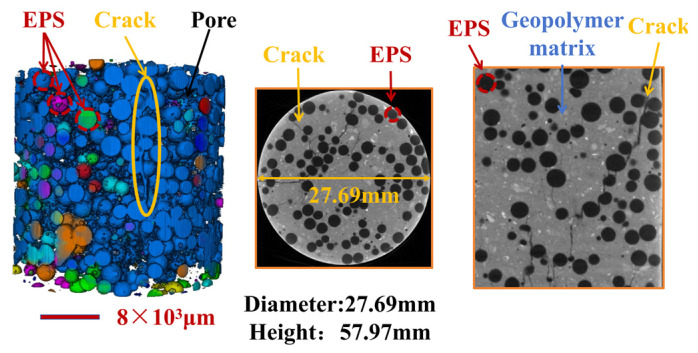
Pore characteristics and EPS particle distribution of LEGC30BF08 specimen.

This study utilised standard cylindrical specimens with dimensions of Φ50×100 mm and conducted quasi-static axial compression tests under displacement control mode with a loading rate of 0.5 mm/min. Additionally, X-ray CT was used for internal pore imaging analysis. Figure 8 shows the 3D reconstruction of the pore characteristics (including the spatial distribution of EPS particles) for the LEGC30BF08 specimen. Incorporating basalt fibres led to a gradient dispersion of EPS particles, effectively mitigating EPS particle migration and segregation.

**Figure 9:**
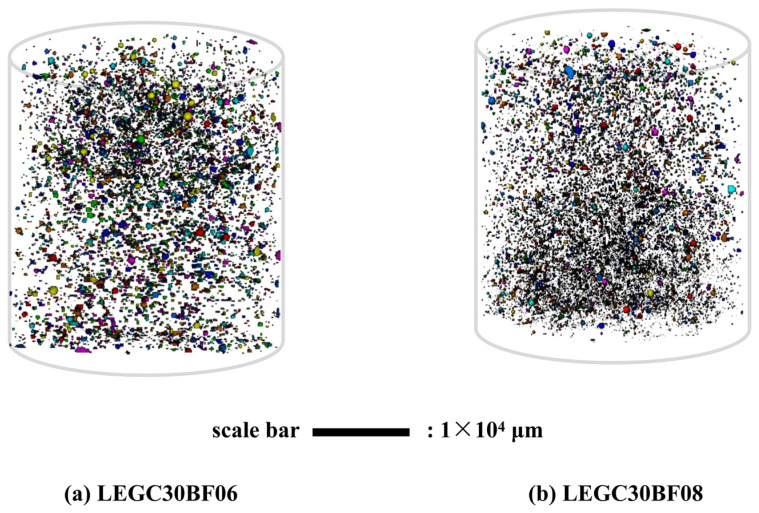
Pore characteristics of selected samples without Expanded Polystyrene (EPS).

By comparing the pore distribution images of the LEGC30BF06 and LEGC30BF08 specimens in Figure 9a,b, the latter exhibits a markedly higher microscopic porosity and stronger pore connectivity, characterised by an increased number density of small pores and the formation of pore clusters penetrating along the specimen height. This difference mainly results from three factors: first, the hydrophobic surface of basalt fibres tends to promote the development of interfacial transition zone (ITZ) pores; second, when the fibre volume fraction exceeds 0.6%, the uniformity of fibre dispersion decreases, leading to obvious fibre clustering, entrapped air, and local pore coalescence, which together induce pore-through effects; and third, the low strength and poor bonding of EPS particles provide additional potential sites for ITZ defects. Under load, such connected pore networks act as incubators for microcrack initiation and propagation, triggering a chain-like crack evolution process (initiation–growth–penetration) and accelerating damage accumulation. When the fibre volume fraction reached 0.8%, the excessive incorporation of basalt fibres further increased the overall specimen porosity, causing the strength loss of the geopolymer matrix to outweigh the bridging and toughening effects of the fibres, thus becoming the dominant mechanism of strength degradation.

This multiscale correlation analysis revealed the underlying mechanism responsible for the deterioration in the mechanical characteristics of the prepared samples when the BF content reached 0.8%. Both compressive and flexural strengths exhibited a similar strength reduction effect at this fiber dosage. In this study, the so-called “multiscale synergistic effect” refers to mutually reinforcing mechanisms across different scales: at the macro scale, DIC indicates a more ductile failure response at the optimal BF content of 0.6%; at the micro scale, SEM confirms the formation of a three-phase continuous load-bearing network (matrix–fiber–EPS/ITZ) that provides effective bridging and anchorage; and at the meso/3D pore scale, CT shows that BF contents above 0.6% (e.g., 0.8%) lead to fiber clustering and pore-through channels, undermining matrix continuity. Taken together, these findings explain the significant strengthening and toughening at 0.6% and the deterioration observed at 0.8%.

To explicitly link microstructural features with macroscopic behavior, CT-derived parameters were quantitatively correlated with the mechanical responses and the observed failure modes. In particular, the pore volume fraction and characteristic pore size obtained from the reconstructions were used to interpret the trends in compressive and flexural strength, as higher porosity and coarser pores disrupt load-bearing continuity and intensify stress concentrations. By contrast, SEM observations confirmed that basalt-fiber (BF) networks provide crack-bridging and pull-out energy dissipation, thereby improving post-peak toughness and splitting resistance at near-optimal fiber contents, and partially compensating for EPS-induced porosity in EPS-rich mixtures.

## 4. Effect of Combined EPS and Basalt Fiber Contents on the Physical Properties of LEGC

### Density and Flowability

**Figure 10:**
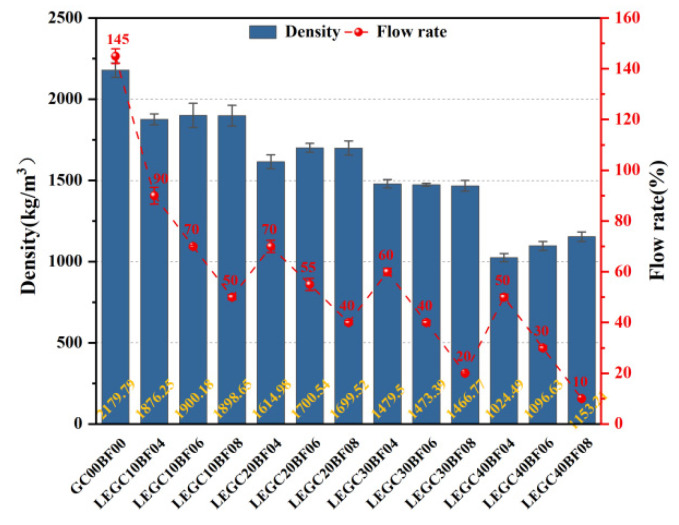
Density and flowability variations of LEGC specimens with varying EPS and BF contents.

Figure 10 presents the variation trends of density and flowability for different LEGC specimens. Overall, although the water-to-binder ratio and superplasticizer (SP) dosage remained constant, the workability of the mixtures decreased markedly with increasing EPS volume fraction. This behavior can be attributed to several factors: (i) the higher EPS content reduces the effective paste volume available for lubrication and dispersion per unit volume; (ii) the hydrophobic nature of EPS particles results in poor wetting by the alkaline geopolymer matrix, thereby increasing interfacial friction; and (iii) when combined with basalt fibers (BF), the overall specific surface area and fiber network effect are enhanced, capturing part of the mixing liquid and reducing the free water available for lubrication. These combined effects increase the mixture’s yield stress and highlight the significant influence of the synergistic action of EPS and fibers on the physical properties of LEGC.

In terms of density, the control specimen without EPS and BF (GC00BF00) exhibited the highest value at 2179.79 kg/m^3^. When the EPS volume content increased to 10% (LEGC10 series), the density dropped significantly to the range of 1876.25–1898.65 kg/m^3^, representing a reduction of approximately 12–14%. Further increasing the EPS content to 20% (LEGC20 series) reduced the density to 1614.98–1699.52 kg/m^3^, with a maximum reduction of up to 25.91%. As the EPS content continued to rise to 30% and 40% (LEGC30 and LEGC40 series), the density further decreased to a range of 1479.50–1153.21 kg/m^3^. The lowest density was recorded for LEGC40BF04 at only 1024.49 kg/m^3^, marking a reduction of over 53% compared to the control group. This trend clearly demonstrates that the incorporation of EPS particles as lightweight aggregates significantly reduces the overall mass of LEGC, whereas the addition of basalt fibers has minimal effect on density.

Flowability decreased with increasing EPS content and, at fixed EPS, declined monotonically with BF volume fraction. The reference mix (GC00BF00) exhibited a flowability of 145%. For BF = 0.4/0.6/0.8%, the values for LEGC10/20/30/40 were 90/70/60/50%, 70/55/40/30%, and 50/40/20/10%, respectively. Accordingly, higher EPS and BF jointly diminish workability: the fibre network increases internal friction and constrains paste mobility, despite its toughening benefits.

## 5. Development and Analysis of Predictive Models for Splitting and Flexural Strengths Based on Compressive Strength

### 5.1. Comparative Analysis of Predictive Models for Splitting Tensile Strength

**Figure 11:**
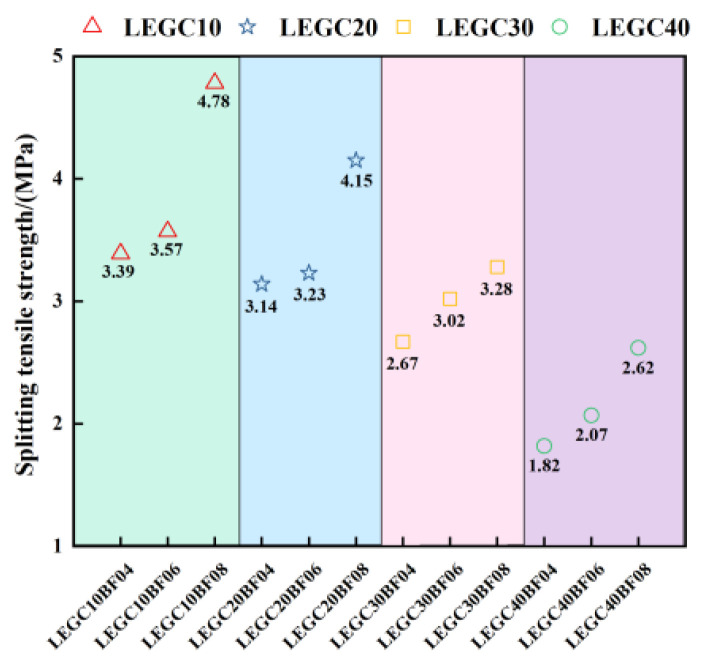
Splitting tensile strength of LEGC specimens with varying EPS and BF contents.

Figure 11 illustrates the variation in splitting tensile strength of LEGC specimens with different combinations of EPS particle and basalt fiber contents. Overall, as the EPS volume fraction increased from 10% to 40%, the splitting tensile strength gradually declined. For instance, the LEGC10BF08 specimen exhibited a splitting tensile strength of 4.78 MPa, whereas LEGC40BF08 reached only 2.62 MPa, reflecting a reduction of 45.19%. This trend aligns with previous studies and is primarily attributed to the inherently low strength of EPS particles compared to traditional aggregates, which results in the formation of numerous weak interfacial zones within the matrix, thereby compromising the tensile performance of the composite [23].

Under a constant EPS content, the inclusion of basalt fibers significantly improved the splitting tensile strength. Taking the LEGC10 series as an example, increasing the fiber volume fraction from 0.4% to 0.8% raised the splitting strength from 3.39 MPa to 4.78 MPa, an enhancement of approximately 40.99%. Compared to 0.6% fiber dosage (3.57 MPa), the increase was 33.89%. These results confirm that the bridging effect of basalt fibers can effectively suppress crack propagation and enhance the cracking resistance of the composite.

However, despite the evident reinforcement effect of basalt fibers, it could not fully offset the strength reduction caused by the incorporation of EPS particles. In the LEGC20, LEGC30, and LEGC40 series, even at the highest fiber dosage of 0.8%, the splitting tensile strengths remained significantly lower than those of the lower-EPS groups. For example, the splitting strength of LEGC40BF08 was only 2.62 MPa, which is markedly lower than that of LEGC10BF08 (4.78 MPa) and LEGC20BF08 (4.15 MPa).

In summary, the influence of EPS and BF in LEGC presents a clear antagonistic relationship. The strength reduction induced by EPS is mainly due to its low modulus and poor mechanical integrity, whereas the reinforcing effect of basalt fibers relies on their proper dispersion and the quality of fiber–matrix interfacial bonding.

**Figure 12:**
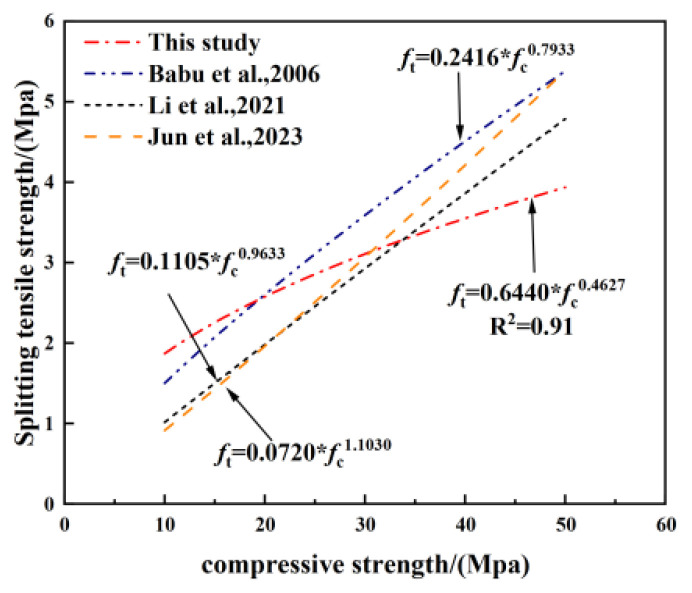
Comparison of predictive models for splitting tensile strength based on compressive strength.

Babu et al. [23,25,32,33,35] developed a set of empirical frameworks to characterise the correlation between $f_{t}$ and $f_{c}$ of lightweight concrete. However, these models do not precisely capture the correlation between $f_{t}$ and quasi-static $f_{c}$ of the LEGC specimens, as illustrated in Figure 12. To address this issue, the current study refines existing empirical models and a new predictive equation for splitting tensile strength based on compressive strength was proposed, as shown in Equation (1).

(1) $$f_{t}=0.0510f_{c}^{1.1520}$$

where $f_{c}$ and $f_{t}$ represent the compressive strength and splitting tensile strength, respectively.

As shown in Figure 12, the proposed empirical model reasonably captures the variation in splitting tensile strength and compressive strength of the LEGC specimens. However, as the splitting specimens in this study were not of standard dimensions, additional testing was necessary to fine-tune and validate Equation (1).

### 5.2. Unified Predictive Model for Flexural and Compressive Strengths with Accuracy Verification

**Figure 13:**
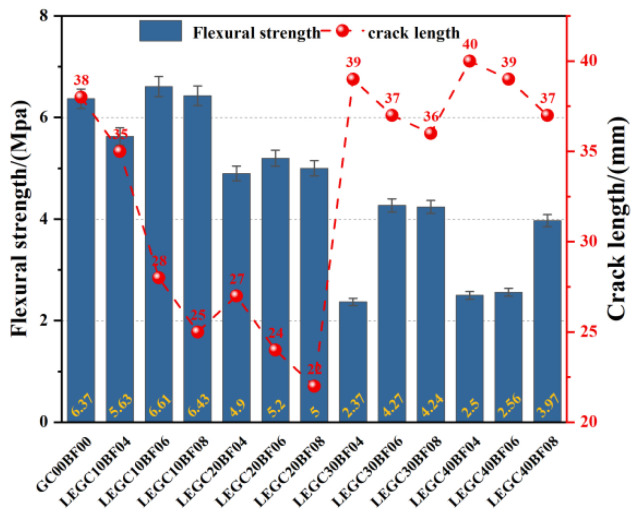
Comparison of flexural strength and final crack length of LEGC specimens with varying BF and EPS contents.

Figure 13 presents the flexural strength and crack development patterns of selected LEGC specimens incorporating various basalt fiber (BF) and EPS particle contents. It can be observed that under a constant EPS volume fraction, the flexural strength of the specimens first increased and then decreased with the increase in BF content. For example, the flexural strengths of LEGC10BF04, LEGC10BF06, and LEGC10BF08 were 5.63 MPa, 6.61 MPa, and 6.43 MPa, respectively. This trend is primarily attributed to the fact that an appropriate amount of basalt fiber (e.g., 0.6% by volume) can bond well with the geopolymer matrix, enabling effective load transfer and crack bridging. However, excessive fiber addition tends to cause fiber agglomeration within the matrix, resulting in multiple weak zones. Under external loading, these weak regions are more likely to fail successively and coalesce into a major crack plane, thus reducing the overall tensile strength of the specimen.

Experimental data showed that as the fiber volume fraction increased from 0.4% to 0.8%, the crack propagation path length in LEGC10 specimens at failure was reduced from 35 mm to 25 mm, a reduction of approximately 28.6%. Microstructural observations further revealed that basalt fibers enhanced the bonding performance of the interfacial transition zone (ITZ) between EPS particles and the geopolymer matrix primarily through a crack-bridging mechanism. Specifically, the hydroxyl groups present on the basalt fiber surface can form chemical bonds with the silicon–oxygen network of the geopolymer matrix, increasing interfacial adhesion. When cracks initiate and propagate under load, these fibers act as bridges, redistributing stresses and limiting crack opening. This bridging effect delays interfacial debonding and restrains microcrack growth, thereby enhancing the integrity of the ITZ. Additionally, the rough surfaces of basalt fibers promote mechanical interlocking with the geopolymer gel phases, further strengthening the bond and mitigating stress concentration. These mechanisms collectively contribute to improved load transfer and reduced premature failure along the fiber–matrix interface.

To ensure the accuracy and comparability of crack length measurements, we recognize that variations can occur across different cross-sections of the same specimen due to material heterogeneity and the nonuniformity of crack propagation paths. Therefore, the “crack length” reported in Figure 13 refers to the average main crack path length obtained from measurements taken on multiple representative cross-sections of each specimen. After the flexural tests, several typical cross-sections were selected along the fracture surface, and the crack lengths were quantified using image analysis software. The arithmetic mean of these values was then reported, which effectively minimizes local fluctuations and provides a more reliable representation of the overall crack propagation behavior of the material.

**Figure 14:**
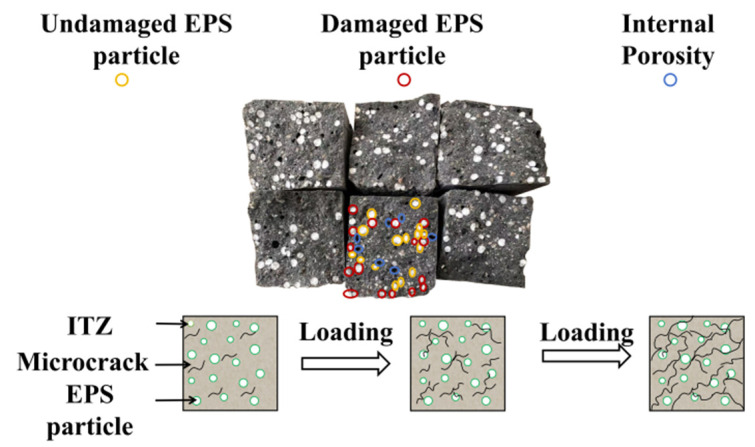
Schematic illustration of the internal damage mechanism of LEGC10BF08 under loading.

The fracture morphology analysis from the flexural strength test, as illustrated in Figure 14, reveals that the LEGC10BF08 specimen exhibited a uniformly distributed three-dimensional EPS particle network within the matrix. Microstructural observations indicate that, under flexural loading, part of the EPS particles experienced intergranular shear failure, while a small proportion underwent interfacial debonding and pull-out behavior. These distinct failure modes interacted synergistically with the geopolymer matrix, contributing to an integrated load transfer mechanism.

From a fracture mechanics perspective, the high compressibility of the EPS particles enhanced the tortuosity index of the crack propagation path, thereby facilitating a shift in the failure mode from brittle fracture to a ductile energy-dissipating mechanism.

Figure 14 also presents a schematic diagram of the damage evolution mechanism in LEGC10BF08. Initially, EPS particles are embedded within the geopolymer matrix, forming interfacial transition zones (ITZ) that contain inherent microcracks. With the application of external loads, these microcracks progressively grow, and stress concentrations develop around the EPS particles, promoting further crack initiation.

As loading continues, the microcracks coalesce and expand, eventually forming penetrating macrocracks that lead to structural failure. The inclusion of EPS alters the crack propagation trajectory by either deflecting cracks around the particles or guiding them along the ITZ. This phenomenon highlights the critical role of ITZ integrity and microcrack evolution in governing the flexural strength and overall failure mechanism of EPS-based geopolymer lightweight concrete.

Jun et al. [25] reported a comparable observation. Building on prior research, the following empirical formula is suggested:

(2) $$f_{r}=0.55f_{c}^{0.6449}$$

**Figure 15:**
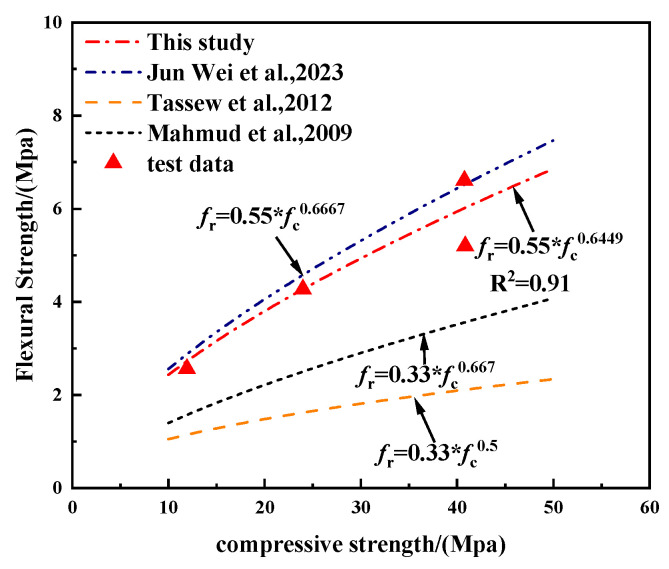
Comparison of fitted curves between flexural and compressive strengths of LEGC based on present study and previous literature data.

As shown in Figure 15, the flexural strength of fiber-reinforced geopolymer concrete increases nonlinearly with compressive strength. The regression model developed in this study demonstrates excellent agreement with the experimental data, yielding a high coefficient of determination (R^2^ = 0.91), which reflects strong correlation. Compared with existing empirical models proposed by Mahmud et al. (2009), Tassew et al. (2012), and Jun Wei et al. (2023) [25,36,37], the present model exhibits superior prediction accuracy, particularly in the high-strength range, where conventional models tend to underestimate the flexural capacity of the material. These findings further confirm the reinforcing effect of basalt fibers on flexural performance and suggest that the proposed model holds significant potential for application in structural design.

**Figure 16:**
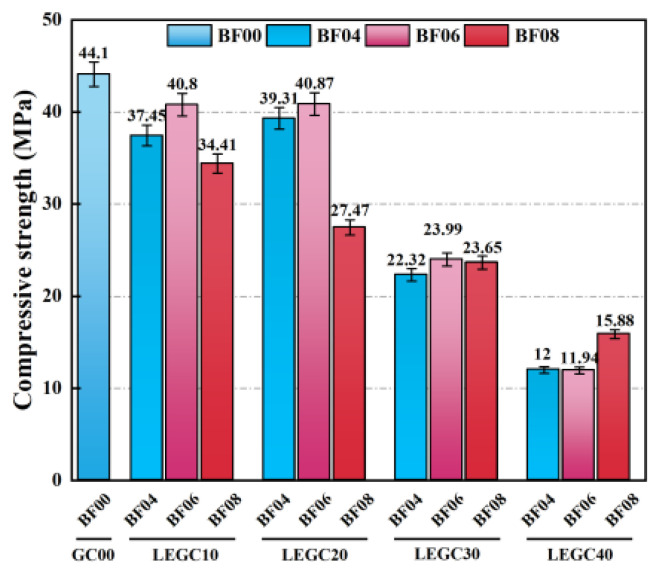
Compressive strength of plain GC and LEGC.

Figure 16 presents the compressive strength results of both conventional geopolymer concrete (GC) and LEGC specimens with varying EPS volume fractions. A consistent decline in compressive strength was observed with the increasing EPS content in LEGC specimens. The compressive strength of the reference GC specimen was 44.1 MPa. When the basalt fiber content was fixed at 6%, the LEGC specimens with 10%, 20%, 30%, and 40% EPS replacements exhibited compressive strengths of 40.80 MPa, 40.87 MPa, 23.99 MPa, and 11.94 MPa, respectively. Compared to the reference GC, these values represent reductions of 7.48%, 7.32%, 45.60%, and 72.93%, respectively. This decline can be attributed to the low mechanical strength of EPS particles and the weakened ITZ formed between the EPS and geopolymer matrix.

Notably, when 40% of the natural fine aggregate by volume was replaced with EPS particles, the compressive strength decreased by 72.93%. This significant reduction is likely due to the inherently low strength of EPS particles and their weak interfacial bonding with the geopolymer matrix. However, when the EPS content was limited to 20% and supplemented with 0.6% basalt fibers (by volume), the resulting LEGC specimen exhibited a density of 1747.6 kg/m^3^ and a compressive strength exceeding 17 MPa, which satisfies the requirements for structural lightweight concrete as specified by ACI 213R-14 [38]—namely, a density between 1120 and 1920 kg/m^3^ and a minimum compressive strength of 17 MPa.

Mechanical results showed that compressive and flexural strengths increased up to 0.6% BF before declining, while splitting tensile strength rose continuously with fiber content. This divergence arises from fiber reinforcement mechanisms, loading modes, and microstructural effects. At 0.6% BF, well-dispersed fibers bridged cracks and enhanced ductility, improving compressive and flexural performance [4]. At 0.8%, fiber clustering and poor orientation reduced workability, increased porosity, and degraded the ITZ, leading to strength loss [39,40]. Compressive and flexural tests, sensitive to matrix compactness, therefore declined, whereas splitting tensile strength—dominated by crack bridging along the fracture plane—continued to increase [15,16].

EPS further weakened the matrix by creating interfacial defects, amplifying the negative effects of high fiber dosages. As a result, compressive and flexural strengths peaked earlier [41], while splitting tensile strength remained less affected by global heterogeneity and sustained an upward trend. In summary, the competing effects of fiber reinforcement and porosity explain the peak at 0.6% BF in compressive and flexural strength, while the monotonic rise in splitting tensile strength underscores the dominant role of localized crack bridging. Thus, 0.6% BF is identified as the optimal dosage for balancing mechanical enhancement and workability in LEGC.

## 6. Development and Validation of a Dual-Factor Predictive Model for the Mechanical Properties of LEGC Based on EPS and Basalt Fiber Dosages

### 6.1. Analysis of Influencing Factors on the Splitting Tensile Performance of LEGC

To systematically investigate the coupled effects of EPS particles and BF content on the splitting tensile strength of fiber-reinforced LEGC, a theoretical framework was established in this study based on ASTM C496/C496M-17 [32]. The standard formula for calculating the splitting tensile strength of conventional concrete is expressed as follows:

(3) $$f_{t}=\frac{2P}{\pi LD}$$

where $f_{t}$ denotes the splitting tensile strength (MPa); P is the maximum applied load at failure (N); D and L represent the diameter and height of the specimen (mm), respectively.

However, for geopolymer concrete incorporating EPS particles and BF, the internal structure becomes more complex and heterogeneous, which renders the conventional formula insufficient to accurately capture its mechanical behavior. Therefore, correction factors must be introduced to account for these effects and to better reflect the actual tensile performance.

Considering the porosity-inducing effect of EPS and the crack-bridging and toughening contribution of basalt fibers, two empirical correction coefficients are introduced: $K_{EPS}$, reflecting the influence of the EPS volume fraction, and $K_{BF}$, accounting for the effect of basalt fiber content. Based on these parameters, a modified predictive model for splitting tensile strength is proposed as follows:

(4) $$f_{t}=f_{t0}\times K_{EPS}\times K_{BF}$$

where $f_{t0}$ denotes the reference splitting tensile strength of the control specimen without EPS and BF additions.

To quantify the adverse effect of EPS incorporation on tensile strength, a negative exponential function is employed to characterize the degradation trend. This model accurately captures the concave-downward decrease in strength and the cumulative effect of weak ITZs, indicating that with increasing EPS content, the integrity of the material undergoes accelerated attenuation. This approach reflects the exponential decay of matrix integrity with rising EPS volume fraction, as induced by the low stiffness and poor interfacial bonding of EPS particles.

(5) $$K_{EPS}=\exp\left(-a\cdot {V^{b}}_{EPS}\right)$$

To quantify the toughening contribution of BF, a linear gain model is introduced, expressed as: in practice, a linear-plus-quadratic expression is adopted to characterize the bridging-induced toughening behavior, which initially increases and then decreases or saturates (i.e., improvement at low dosages, while excessive addition causes clustering and non-uniform dispersion, leading to diminishing marginal benefits).

(6) $$K_{BF}=\left(1+cV_{BF}+dV_{BF}^{2}\right).$$

where $V_{BF}$ and $V_{BF}$ represent the volume fractions of EPS particles and basalt fibers. a, b, c, and d are empirically derived fitting coefficients.

By substituting the above correction factors into Equation (4), the final predictive model for splitting tensile strength is established.

(7) $$f_{t}=f_{t0}\cdot \exp\left(-a{V^{b}}_{EPS}\right)\cdot \left(1+cV_{BF}+dV_{BF}^{2}\right)$$

Based on regression analysis of the experimental data, the optimal fitting parameters were determined as a = 1.34 × 10^–4^, b = 2.28, c = −1.5, and d = 1.74. These parameters capture the coupled influence of EPS content and basalt fiber dosage on the splitting tensile strength of LEGC. Specifically, a represents the sensitivity of strength to EPS content; b and c describe the reinforcing effect of the fibers and the weakening effect of EPS, respectively; and d is a correction factor reflecting the matrix–fiber synergy. As EPS content increases, the splitting tensile strength decreases exponentially due to reduced matrix continuity and weaker ITZ bonding. At a moderate fiber content (0.6%), the bridging effect of the fibers partially offsets this strength loss, whereas at a higher fiber content (0.8%), fiber clustering and increased porosity diminish the reinforcement.

Consequently, the final expression for the predicted splitting tensile strength is given by:

(8) $$f_{t}=5.20\cdot \exp\left(0.000134V_{EPS}^{2.28}\right)\cdot \left(1-1.5V_{BF}+1.74V_{BF}^{2}\right)$$

**Figure 17:**
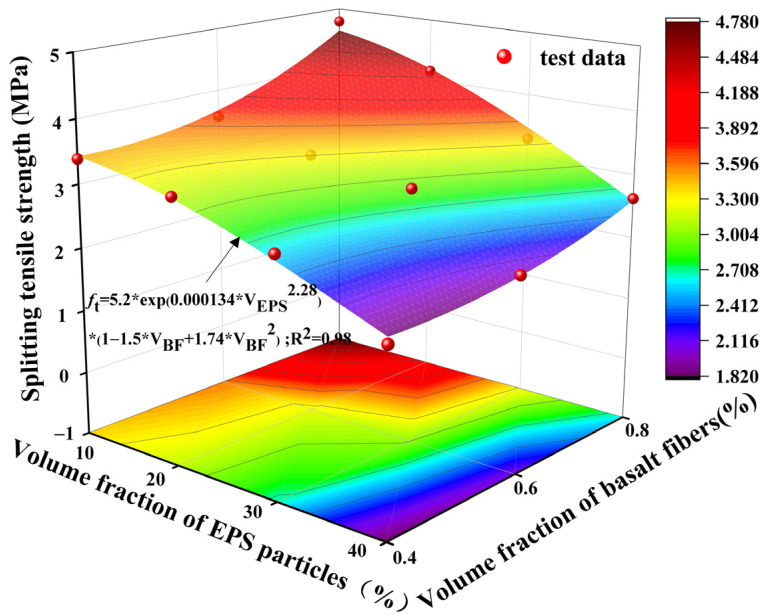
3D fitting surface of LEGC splitting tensile strength with EPS and BF content.

Figure 17 presents the 3D fitted surface for the splitting tensile strength prediction, illustrating the coupled influence of EPS particle volume content and basalt fiber (BF) volume fraction. The results clearly demonstrate a nonlinear interactive relationship between the two variables. Overall, an increase in EPS content leads to a continuous decline in splitting tensile strength, whereas the incorporation of BF effectively counteracts this adverse effect, resulting in a net strengthening behavior.

In terms of EPS influence, the splitting tensile strength decreases progressively as the EPS volume increases from 10% to 40%. This reduction is primarily attributed to the low density and poor mechanical strength of EPS particles, which increase internal porosity and weaken the ITZ between particles and matrix. These effects collectively compromise the material’s tensile resistance. The regression surface shows a pronounced concave profile along the EPS axis, highlighting the intensifying negative effect at higher EPS contents.

In contrast, the incorporation of BF markedly improves crack resistance and toughness. As the fiber volume fraction increases from 0.4% to 0.8%, splitting tensile strength continues to rise, reflecting the effectiveness of fiber bridging. In this study, “toughness enhancement” refers to increased energy absorption and post-peak load-bearing capacity. At low-to-moderate EPS contents and around 0.6% BF, residual capacity and energy absorption are significantly improved. However, at high EPS levels (≥30%) or with 0.8% BF, post-peak softening becomes unstable due to fiber clustering and increased ITZ porosity, consistent with the bridging–clustering competitive mechanism observed in SEM and CT. Overall, the compensatory role of fibers is more pronounced at higher EPS contents.

Overall, the combined effect of EPS and BF on tensile strength is not purely additive but exhibits a complex synergistic interaction. The optimal performance is achieved at a moderate EPS content (around 20%) and a BF dosage exceeding 0.6%, validating the rationality of the dual-factor prediction model proposed in this study. The regression model achieves a high coefficient of determination (R^2^ = 0.98), confirming its accuracy and predictive capability. These findings provide valuable guidance for the performance-oriented mix design of fiber-reinforced lightweight geopolymer composites.

### 6.2. Model Development for Flexural Performance Response

To quantitatively investigate the coupled effects of EPS particle content and BF volume fraction on the flexural strength of LEGC, an empirical predictive model was developed using the response surface methodology (RSM). The model construction process involved a systematic derivation of the nonlinear interaction mechanisms between the influencing variables.

#### 6.2.1. Model Selection and Variable Definition

Given the nature of the experimental design and the response characteristics, a quadratic response surface model was adopted as the baseline framework. This model includes linear, quadratic, and interaction terms of the independent variables, enabling effective characterization of both nonlinear enhancement and weakening effects. The general form of the model is expressed as follows:

(9) $$f_{r}=a+b\cdot V_{\mathrm{EPS}}+c\cdot V_{\mathrm{BF}}+d\cdot V_{EPS}^{2}+e\cdot V_{BF}^{2}+f\cdot V_{\mathrm{EPS}}V_{\mathrm{BF}}$$

In the above model, $f_{r}$ denotes the flexural strength of LEGC (MPa); $V_{\mathrm{EPS}}$ and $V_{\mathrm{BF}}$ represent the volume fractions of EPS and basalt fiber (%), respectively. The coefficients a, b, c, d, e, f are the fitting parameters that quantify the relative influence of each term on the response variable.

The model incorporates three physical mechanisms: the linear terms (b,c) capture the direct reinforcing or weakening effects of EPS and BF on the composite; the quadratic terms (d,e) describe nonlinear enhancement or degradation behavior; and the interaction term (f) quantifies the synergistic or antagonistic interplay between EPS particles and basalt fibers. This formulation enables a comprehensive understanding of how the two constituents influence the flexural performance of geopolymer lightweight composites under different mix designs.

#### 6.2.2. Interaction Effect Analysis and Physical Interpretation of Variables

Based on the experimental data, increasing the EPS content leads to a reduction in the effective load-bearing area of the matrix and a deterioration of the interfacial transition zone (ITZ), thereby causing a significant decline in flexural strength. This reveals a negative correlation between EPS dosage and flexural performance.

(10) $$\frac{\partial f_{r}}{\partial V_{\mathrm{EPS}}}=b+2dV_{\mathrm{EPS}}+fV_{\mathrm{BF}}<0$$

Conversely, the appropriate incorporation of basalt fibers (BF) enhances crack-bridging and energy dissipation capacities, thus improving the flexural strength of the composite within a reasonable dosage range.

(11) $$\frac{\partial f_{r}}{\partial V_{\mathrm{BF}}}=c+2eV_{\mathrm{BF}}+fV_{\mathrm{EPS}}>0$$

However, when the fiber content exceeds the optimal threshold, agglomeration and uneven dispersion may occur, compromising the reinforcing efficiency and even resulting in a reduction in flexural strength, as indicated by a negative coefficient in the predictive model e < 0.

#### 6.2.3. Regression Coefficient Estimation and Model Validation

A quadratic response surface regression was performed on the experimental data to estimate the coefficients a through f, resulting in the following empirical model:

(12) $$f_{r}=6.82-0.135V_{\mathrm{EPS}}+2.94V_{\mathrm{BF}}-0.0041V_{EPS}^{2}-3.18V_{BF}^{2}-0.018V_{\mathrm{EPS}}V_{\mathrm{BF}}$$

**Figure 18:**
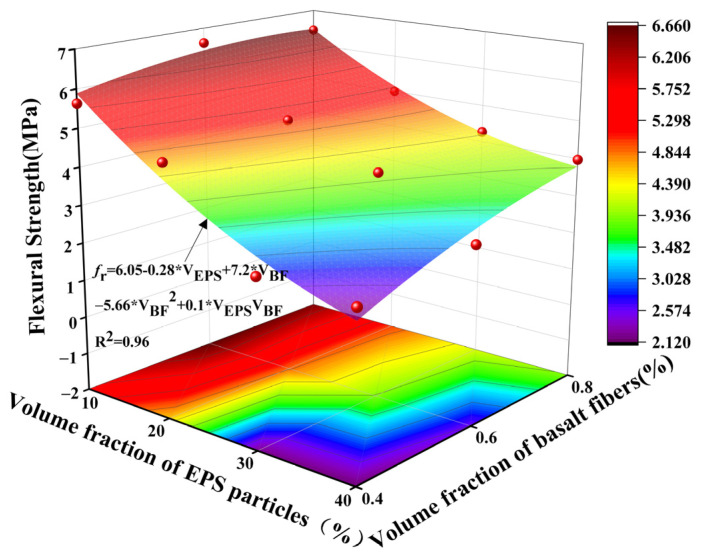
3D fitting surface of LEGC Flexural strength with EPS and BF content.

The regression analysis yielded a coefficient of determination (R^2^) of 0.96, as shown in Figure 18, indicating a high degree of fit between the model and the experimental data. This suggests that the proposed model effectively captures the influence of both EPS and basalt fiber (BF) contents on the flexural strength of LEGC.

Beyond accurately reflecting the synergistic and competing interactions between EPS and BF, the model also provides practical guidance for optimizing mix proportions in structural design. Specifically, the optimal flexural performance was achieved when the EPS volume fraction was maintained between 10% and 20% and the BF content was approximately 0.6%. However, when the EPS content exceeded 30%, the positive contribution of BF became marginal, even with increased fiber dosage, highlighting the need to further refine the particle size and dispersion characteristics of EPS aggregates.

In summary, the proposed constitutive model integrates empirical observations with physical mechanisms, offering strong predictive and explanatory capabilities. It provides a theoretical foundation for regulating the mechanical behavior of EPS–BF hybrid geopolymer composites.

### 6.3. Nonlinear Regression Modeling of Compressive Strength and Evaluation of Coupled Effects of EPS and BF Content

The incorporation of Expanded Polystyrene (EPS) particles and basalt fibers (BF) significantly affects the compressive strength of LEGC specimens. Roy et al. [42], based on extensive experimental investigations on EPS concrete, systematically evaluated the influence of EPS particle size and volume fraction on mechanical strength. Employing a porosity-based analytical approach, they treated EPS inclusions as voids within the cementitious matrix, neglecting their elastic contribution. Accordingly, a strength prediction model was proposed, which correlates the compressive strength of EPS concrete with the particle size and volumetric content of the EPS aggregates. As shown in Equation (13):

(13) $$f_{c}=\left(\frac{\alpha \left(1-\frac{p}{p_{\max}}\right)}{\alpha +\frac{p}{p_{\max}}}\right)f_{cm}$$

where $f_{c}$ denotes the compressive strength of the geopolymer lightweight concrete, $f_{cm}$ is the compressive strength of the plain matrix, p is the volume fraction of EPS particles, $p_{\max}$ represents the maximum porosity, and α is a fitting coefficient.

Based on CECS 38-2004 [43], Jun reported that the compressive strength of conventional concrete exhibits an approximately linear relationship with the volume content of basalt fibers. Building upon Roy’s strength prediction model, a modified formulation was developed that incorporates the effects of EPS particle size, EPS volume fraction, and basalt fiber volume content.

(14) $$f_{c}=\beta \left(\frac{\alpha \left(1-\frac{p}{p_{\max}}\right)}{\alpha +\frac{p}{p_{\max}}}\right)f_{cm}\left(1+\gamma \frac{l}{d}\varphi \right)$$

where β and α denote the influence coefficients of the LEGC matrix and basalt fibers, respectively; l represents the length of the basalt fiber, d is its diameter, and φ corresponds to the volume fraction of the basalt fibers.

To gain deeper insight into the synergistic regulatory mechanisms of combined EPS particle and basalt fiber incorporation on the compressive strength of LEGC specimens, a nonlinear coupled regression model was developed based on existing models. This model integrates the effects of EPS particle size, volumetric content, and fiber dimensional parameters. Considering the heterogeneous synergy in which EPS particles act as a lightweight weakening phase and basalt fibers serve as a reinforcing phase, the following predictive model is proposed:

(15) $$f_{c}=-0.08624\left(\frac{-256.56-p}{14.69+p}\right)44.1\left(1+0.04692\varphi \right)$$

The compressive strength of the geopolymer matrix was determined to be 44.1 MPa; therefore, the value of $f_{cm}$ was set to 44.1 MPa. The basalt fibers used in this study had a diameter and length of 15 μm and 9 mm, respectively; thus, d= 15 μm and l = 9 mm, respectively. The p from 0 to 0.3, while φ from 0.004 to 0.008.

**Figure 19:**
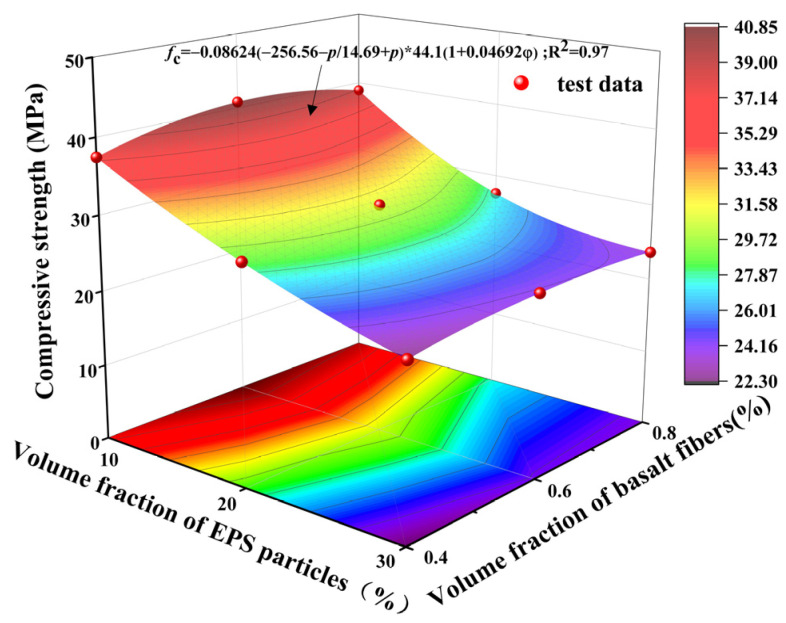
Comparison between the predicted compressive strength by Equation (15) and experimental results.

According to the response surface fitting results shown in Figure 19, the proposed model achieved a high coefficient of determination (R^2^ = 0.97), indicating satisfactory predictive performance and its ability to reasonably capture the nonlinear coupling characteristics between EPS and BF. As observed from the surface trend, increasing the EPS content significantly reduced the compressive strength, particularly beyond the 20% threshold, where the strength degradation became more pronounced. This suggests a negative influence of EPS particles on matrix densification and mechanical integrity. In contrast, the addition of basalt fibers at an optimal volume fraction (approximately 0.6%) partially offset the strength loss caused by EPS, highlighting the compensatory reinforcement effect of the fibers.

However, due to the high porosity and low elastic modulus of EPS, along with the formation of weak interfacial zones between EPS and the matrix, its adverse effects become dominant at higher dosage levels, thereby limiting the toughening contribution of basalt fibers. Additionally, the model was developed under the assumptions of ideal spherical particles and uniform distribution. Slight underestimations were observed for several high-strength specimens, indicating the need to incorporate shape factor and interfacial correction coefficients in future studies to improve prediction accuracy [44].

## 7. Development of a Novel Coupled Damage Constitutive Model for Splitting Tensile Behavior

### 7.1. Elasto-Plastic Damage Model for Splitting Tensile Behavior of LEGC Based on Energy Dissipation

To accurately characterize the nonlinear mechanical behavior of fiber-reinforced LEGC under tensile loading, a novel elasto-plastic damage constitutive model is proposed in this study. This model incorporates both statistical damage theory and energy dissipation mechanisms, while introducing irreversible post-peak plastic deformation to more realistically simulate the mechanical response of the material.

#### 7.1.1. Helmholtz Free Energy and Effective Stress

Based on the principles of irreversible thermodynamics and continuum damage mechanics, the Helmholtz free energy density function of the system can be expressed as follows:

(16) $$\psi =\frac{1}{2}E_{0}(1-D){\left(\varepsilon -\varepsilon^{p}\right)}^{2}$$

The effective stress expression can be derived by taking the partial derivative of the Helmholtz free energy with respect to the elastic strain.

(17) $$\sigma =\frac{\partial \psi }{\partial \varepsilon }=E_{0}(1-D)(\varepsilon -\varepsilon^{p})$$

where $E_{0}$ denotes the initial elastic modulus, D ∈ [0, 1] is the scalar damage variable, ε represents the total strain, and $\varepsilon^{p}$ denotes the irreversible plastic strain.

#### 7.1.2. Coupled Damage Evolution Law

To simultaneously capture the damage behavior of LEGC resulting from microstructural degradation and energy dissipation, the total damage variable D is modeled as a coupled formulation that integrates both statistical and energy-based damage components:

(18) $$D(\varepsilon )=1-(1-D_{\mathrm{stat}})(1-D_{\mathrm{energy}})$$

The statistical damage model is formulated based on the Weibull distribution:

(19) $$D_{\mathrm{stat}}=1-\exp(-b\varepsilon )$$

while the energy-based damage model is established using a dissipation energy threshold:

(20) $$D_{\mathrm{energy}}=1-\exp\left[-{\left(\frac{\varepsilon }{\varepsilon_{p}}\right)}^{a}\right]$$

where a and b are the damage control parameters, and $\varepsilon_{p}$ represents the peak strain corresponding to tensile failure.

#### 7.1.3. Evolution of Plastic Strain

After the material reaches the peak strain, the bridging and pull-out effects of basalt fibers result in residual deformation. In this study, the evolution of plastic strain is characterized using a power-law formulation:

(21) $$\varepsilon_{p}(\varepsilon )=\left\{\begin{array}{ll} 0, & \varepsilon <\varepsilon_{p}\\ h{\left(\frac{\varepsilon -\varepsilon_{p}}{\varepsilon_{p}}\right)}^{i}\cdot \varepsilon_{p}, & \varepsilon \geq \varepsilon_{p} \end{array}\right.$$

where h is the plastic development coefficient, and i denotes the plastic growth exponent.

#### 7.1.4. Final Constitutive Relationship

By integrating the formulations derived above, the final constitutive stress–strain relationship is established as follows:

(22) $$\sigma (\varepsilon )=E_{0}[1-(1-\exp(-b\varepsilon ))(1-\exp\left(-{\left(\frac{\varepsilon }{\varepsilon_{p}}\right)}^{a}\right))]\cdot (\varepsilon -\varepsilon_{p}(\varepsilon ))$$

### 7.2. Validation of the Damage Constitutive Model

**Figure 20:**
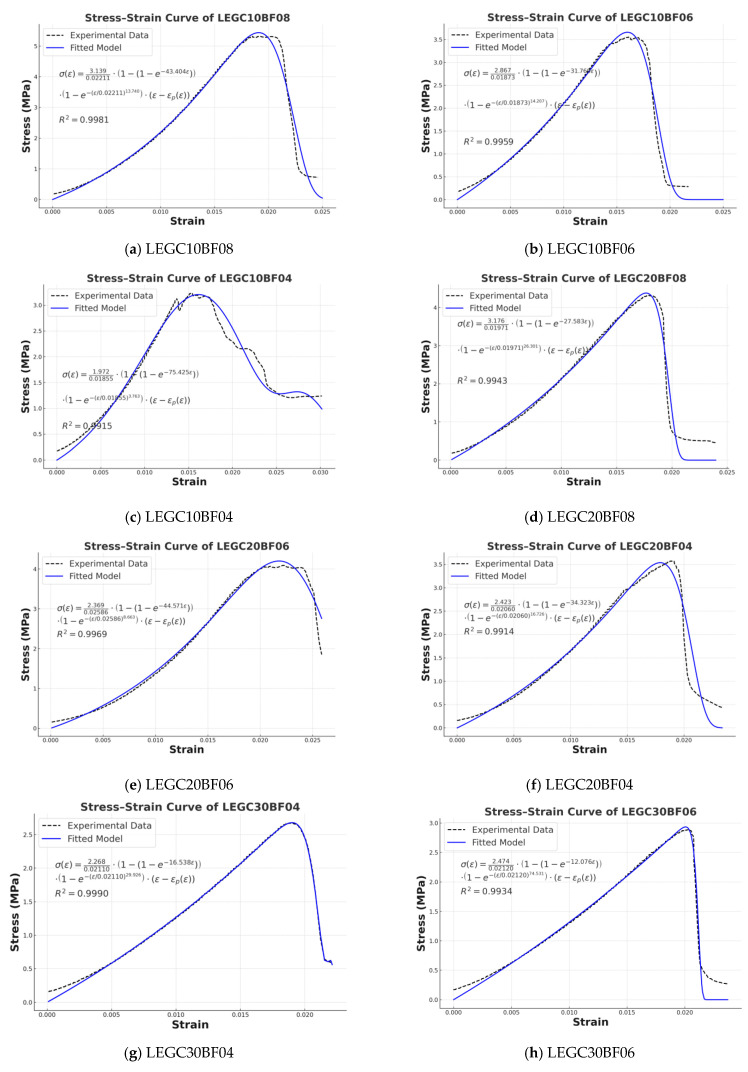

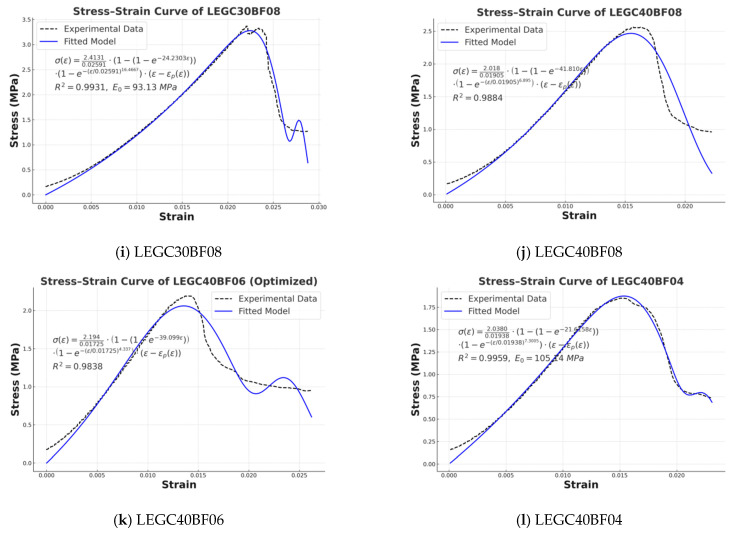
Comparison between experimental and fitted stress–strain curves of different LEGC specimens.

The elastoplastic damage model proposed in Section 6.1 incorporates six fitting parameters, which are determined based on composite damage mechanics using linear regression techniques. Details of the parameter values are presented in Table 6 and Table 7. Figure 20 compares the predicted results from the model with the experimental data, demonstrating the accuracy and applicability of the proposed model.

Note that the figures presenting splitting tensile strength report the group mean of three specimens (n = 3), calculated in accordance with ASTM C496, whereas the stress–strain figures display the response of a single representative specimen along with its corresponding fitted model curve.

The proposed model demonstrated a high level of agreement in predicting the stress–strain responses of LEGC specimens reinforced with varying BF volume fractions (0.4%, 0.6%, and 0.8%), achieving coefficients of determination (R^2^) greater than 0.95. This indicates the model’s robust capability in capturing the nonlinear mechanical behavior of fiber-reinforced geopolymer lightweight concrete.

To evaluate the model’s performance in greater detail, the LEGC10 series was selected as a representative case. The stress–strain curves of specimens with different BF contents exhibited distinct variations, and the fitted results for all groups yielded strong correlations, further confirming the accuracy and applicability of the model in describing the deformation mechanisms of composite materials.

In the ascending branch of the curve, an increase in fiber content led to a progressive rise in peak stress and an enhancement in initial stiffness, reflecting improved load-bearing capacity and crack resistance. Notably, the LEGC10BF08 specimen exhibited the highest peak strength (~5.3 MPa) and the steepest initial slope, indicating a significant improvement in stiffness and resistance to deformation.

In the descending branch, the bridging action of fibers effectively delayed crack propagation, enhancing ductility and energy dissipation capacity. The LEGC10BF04 specimen displayed a typical brittle failure, whereas LEGC10BF06 and LEGC10BF08 showed smoother post-peak softening and appreciable residual load-bearing capacity. These results suggest that an appropriate BF content can significantly improve the post-peak performance of LEGC.

Overall, the fitted model curves exhibit good smoothness and continuity, accurately describing the nonlinear response characteristics of LEGC during both pre-peak hardening and post-peak damage evolution, and demonstrating strong predictive capability and adaptability.

### 7.3. Comparative Analysis of Stress–Strain Characteristics

**Figure 21:**
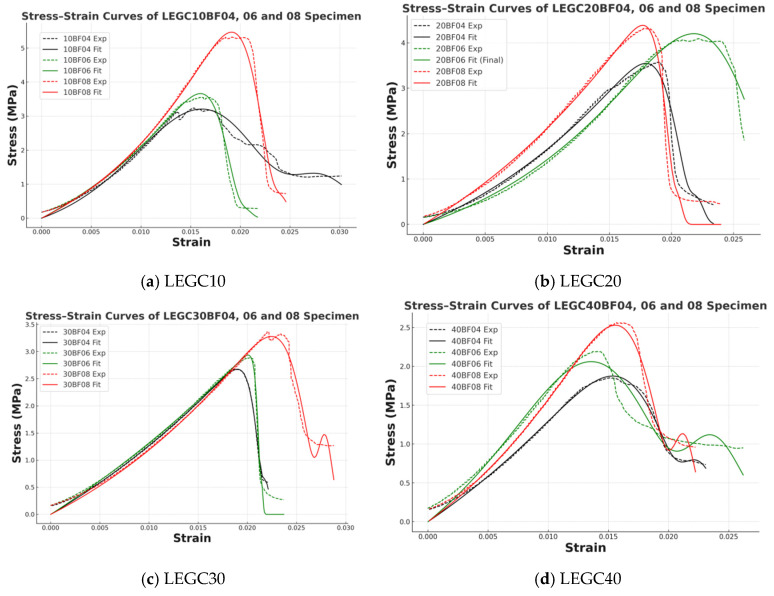
Comparison of experimental and fitted stress–strain curves for LEGC10, LEGC20, LEGC30, and LEGC40 specimens with varying basalt fiber contents.

To systematically investigate the influence of EPS content and BF volume fraction on the mechanical behavior of LEGC, four representative groups of specimens—LEGC10, LEGC20, LEGC30, and LEGC40—were selected for experimental testing and model fitting comparison, as illustrated in Figure 21. The main findings are summarized as follows:

The stress–strain behavior of LEGC was strongly influenced by EPS and BF contents. Increasing EPS from 10% to 40% caused a progressive reduction in peak stress, as matrix dilution and interfacial weakening outweighed fiber reinforcement. For example, LEGC40BF04 reached only 2.04 MPa, much lower than LEGC10BF04 at the same fiber content, highlighting the adverse effect of excessive EPS.

Peak strain followed a rise–fall trend, with LEGC20 showing the most favorable response. LEGC20BF06 exhibited smooth curve transitions and high fitting accuracy (R^2^ > 0.99), indicating stable crack propagation and balanced strength–ductility. In contrast, high-EPS specimens (e.g., LEGC40BF06) displayed unstable post-peak behaviors such as “plateau–rebound” or “sharp drop–residual” softening, associated with pore collapse and ITZ debonding.

At low to moderate EPS levels, fiber reinforcement was more effective. LEGC10 and LEGC20 with 0.6–0.8% BF achieved higher strength and ductility, confirming the crack-bridging and load-transfer roles of basalt fibers.

In summary, EPS and BF jointly govern the mechanical response of LEGC. LEGC20BF06 provided the best balance of strength, ductility, and post-peak stability, while the LEGC40 series, though lightweight, requires further improvement in structural stability.

## 8. Conclusions

This study systematically investigated the influence of basalt fibers and EPS particles on the mechanical properties of LEGC through a series of experimental tests. The results indicate that appropriate incorporation of basalt fibers and EPS can significantly enhance the strength, ductility, and crack resistance of LEGC. Moreover, the optimal dosages play a critical role in maximizing the composite mechanical performance. Empirical prediction models for compressive, flexural, and splitting tensile strength were developed, along with a coupled elastoplastic damage constitutive model, offering theoretical support for the engineering application of LEGC. The main conclusions are as follows:

1. Co-regulation of Density and Workability by EPS and Basalt Fibers

The density of LEGC decreased steadily with increasing EPS content, while flowability was significantly reduced due to the combined effects of EPS and basalt fibers. A well-balanced mixture of EPS and BF is beneficial for achieving a compromise between lightweight properties and acceptable workability.

2. LEGC Exhibits Favorable Load-Bearing Capacity, Meeting Engineering Criteria

Based on optimized formulation (GC00BF00) and ambient curing, the 28-day compressive strength of LEGC specimens ranged from 11.94 MPa to 40.87 MPa, with densities between 1096.63 and 1900.18 kg/m^3^. Notably, the LEGC20BF06 specimen exhibited a compressive strength of 40.87 MPa and a density of 1700.54 kg/m^3^, meeting the structural lightweight concrete criteria outlined in ACI 213R-14. These results demonstrate potential applications in load-bearing components and precast wall panels.

3. Combined Dosage of EPS and Fibers Significantly Affects Failure Modes and Strength Evolution

DIC-3D observations revealed that LEGC exhibits various cracking patterns under splitting tension, including crushing, through-cracks, and edge spalling. Moderate BF content effectively controlled crack propagation and improved both strength and toughness. In contrast, excessive EPS content intensified interfacial weakening and microcrack growth, leading to reduced overall mechanical performance.

4. Consistency Between Macroscale Mechanical Tests and Microscale Structural Characterization

Compression and flexural tests confirmed that moderate basalt fiber dosage improved mechanical performance, while excessive addition led to strength degradation. CT and SEM analyses showed that high fiber content increased porosity and facilitated microcrack development. However, at optimal content (e.g., 0.6%), the synergistic crack-bridging effect of BF and EPS was evident, transitioning the failure mode from brittle to ductile.

5. Proposed Constitutive Model and Empirical Formulas Exhibit High Applicability and Engineering Relevance

The proposed elastoplastic damage model, defined by six fitting parameters, accurately captured the full stress–strain behavior of LEGC and aligned well with experimental results, providing insights into damage evolution mechanisms. The developed predictive formulas for compressive, flexural, and splitting tensile strength effectively characterize performance trends within the investigated density range, offering practical utility for engineering design.

In summary, this research elucidates the synergistic mechanisms of EPS and basalt fibers in influencing the performance of LEGC, and establishes corresponding constitutive and predictive models, providing theoretical support for the application of LEGC in green building materials and prefabricated structures. Nonetheless, limitations remain, such as the lack of systematic quantification of shrinkage behavior and dynamic mechanical properties under ultra-low density. Future work should aim to expand the applicability of the models and further validate them through comprehensive testing.

## Figures and Tables

**Table 1:** Chemical composition of fly ash and slag.

Component	Fly Ash (wt%)	Slag (wt%)
SiO_2_	43	34.00
Al_2_O_3_	23	17.60
CaO	5.6	34.00
MgO	0.95	6.21
Fe_2_O_3_	2.5	1.01
SO_3_	0.8	1.62
Total	75.85	94.44

**Table 2:** Properties of basalt fibre.

Diameter (μm)	Density (kg/m^3^)	Elastic Modulus (GPa)	Tensile Strength (MPa)	Elongation at Break (%)
7–15	2.63–2.65	91–110	3000–4800	3.1

**Table 3:** Parameters of Sodium Silicate Solution.

Na_2_O (%)	SiO_2_ (%)	H_2_O (%)	Ms (SiO_2_/Na_2_O)	Pomerol (Be/68 °F)	Specific Gravity
8.54	27.30	64.16	3.30	38.5	1.37

**Table 4:** Mix Proportion Design of Test Specimens.

Specimen ID	Mix Proportions of Main Materials						EPS		BF		Sp/(kg/m^3^)
	Slag	FA	NaOH	Na_2_SiO_3_	Sand	H_2_O	Wt/(kg/m^3^)	Vol (%)	Wt/ (kg/m^3^)	Vol/(%)	
GC00BF00	348.8	348.8	45.2	216.9	1011.8	118	0	0	0	0	10.5
LEGC10BF04	348.8	348.8	45.2	216.9	811.8	118	1.8	10	10.6	0.4	10.5
LEGC10BF06	348.8	348.8	45.2	216.9	811.8	118	1.8	10	15.9	0.6	10.5
LEGC10BF08	348.8	348.8	45.2	216.9	811.8	118	1.8	10	21.2	0.8	10.5
LEGC20BF04	348.8	348.8	45.2	216.9	611.8	118	3.7	20	10.6	0.4	10.5
LEGC20BF06	348.8	348.8	45.2	216.9	611.8	118	3.7	20	15.9	0.6	10.5
LEGC20BF08	348.8	348.8	45.2	216.9	611.8	118	3.7	20	21.2	0.8	10.5
LEGC30BF04	348.8	348.8	45.2	216.9	411.8	118	5.5	30	10.6	0.4	10.5
LEGC30BF06	348.8	348.8	45.2	216.9	411.8	118	5.5	30	15.9	0.6	10.5
LEGC30BF08	348.8	348.8	45.2	216.9	411.8	118	5.5	30	21.2	0.8	10.5
LEGC40BF04	348.8	348.8	45.2	216.9	211.8	118	7.3	40	10.6	0.4	10.5
LEGC40BF06	348.8	348.8	45.2	216.9	211.8	118	7.3	40	15.9	0.6	10.5
LEGC40BF08	348.8	348.8	45.2	216.9	211.8	118	7.3	40	21.2	0.8	10.5

**Table 5:** Relevant data of the geopolymer lightweight concrete specimens.

No.	Test Description	Dimensions (mm)	Number of Groups	Specimens Per Group	Total Specimens
1	Splitting Tensile Strength Test	50 × 100	13	3	39
2	Flexural and Compressive Strength Test	40 × 40 × 160	13	3	39

**Table 6:** Definitions and Descriptions of Parameters in the Elastoplastic Damage Constitutive Model.

Parameter	Description	Unit
$\sigma_{p}$	Peak stress	MPa
$\varepsilon_{p}$	Peak strain	--
*a*	Energy-based damage index	--
*b*	Statistical damage coefficient	--
$E_{0}$	Initial elastic modulus	MPa
*h*	Plastic strain evolution coefficient	--
*i*	Plastic strain growth exponent	--

**Table 7:** Model Fitting Parameters of LEGC Specimens with Varying EPS and BF Contents.

Specimen ID	Fitted Parameters							
	$\sigma_{p}$	$\varepsilon_{p}$	*a*	*b*	$E_{0}$	*h*	*i*	*R^2^*
LEGC10BF04	1.972	0.01855	3.763	−75.425	106.26	−374.866	5.828	0.9915
LEGC10BF06	2.867	0.01873	14.207	−31.769	153.09	38.037	13.910	0.9959
LEGC10BF08	3.139	0.02211	13.74	−43.404	141.97	−168.38	154.52	0.9981
LEGC20BF04	2.423	0.02060	16.726	−34.323	117.65	−285,787.4	1942.83	0.9914
LEGC20BF06	2.369	0.02586	8.6626	−44.5713	91.6	618,804.4	−3270.8	0.9969
LEGC20BF08	3.176	0.01971	26.301	−27.583	161.14	1444.205	3052.53	0.9943
LEGC30BF04	2.268	0.02110	29.926	−16.538	107.50	−10,588,465	4.6018	0.9943
LEGC30BF06	2.474	0.02120	74.535	−12.076	116.69	−177,214.7	332.374	0.9934
LEGC30BF08	2.413	0.02591	16.467	−24.2303	93.13	−343,671.3	4.1504	0.9931
LEGC40BF04	2.038	0.01938	7.301	–21.616	105.14	–1814.642	3.431	0.9959
LEGC40BF06	2.194	0.01725	4.337	−39.0991	127.19	−995.5579	4.7579	0.9838
LEGC40BF08	2.018	0.01905	6.8949	−41.8101	105.90	−28,932.03	215.139	0.9884

## Data Availability

The original contributions presented in this study are included in the article. Further inquiries can be directed to the corresponding author.
